# Volatility forecasts of stock index futures in China and the US–A hybrid LSTM approach

**DOI:** 10.1371/journal.pone.0271595

**Published:** 2022-07-28

**Authors:** Xue Chen, Yan Hu

**Affiliations:** 1 School of Finance, Southwestern University of Finance and Economics, Chengdu, China; 2 School of Statistics, Chengdu University of Information Technology, Chengdu, China; Aristotle University of Thessaloniki, GREECE

## Abstract

This paper is concerned with the unsolved issue of how to accurately predict the financial market volatility. We propose a novel volatility prediction method for stock index futures prediction based on LSTM, PCA, stock indices and relevant futures. Inspired by the recent advancement of deep learning methodology, six models that combine a variety of artificial intelligence techniques are compared, including ANN, ANN(PCA), ANN(AE), LSTM, LSTM(PCA), and LSTM(AE). That is, in the design and comparison of the proposed AI models, we consider the combination of two dimensionality reduction methods (PCA and AE) and two typical neural networks (ANN and LSTM) in processing time series data. Besides, to further assess the prediction performance of the proposed models, two widely-applied statistical models (i.e. AR and EGARCH) on volatility prediction are used as benchmarks. In the empirical study, we collect financial trading data in both China and the US, and compare the performances of different models in predicting 5 days and 10 days ahead volatilities of stock index futures. In all, our analysis supports the use of LSTM(PCA) model to tackle those irregular and complex datasets.

## 1. Introduction

An important common feature of stock markets across countries is volatility. It is a crucial concept in finance that indicates how the asset prices fluctuate. Practitioners in the financial industry thus have the incentive to develop models that can better predict the market volatility, so as to do better in various aspects such as risk management, financial hedging, portfolio management, as well as derivative pricing. From theoretical perspective, an advanced volatility prediction model that fits the data better can also provide support to better understand the fluctuating market dynamics. Nevertheless, how to accurately predict volatility is basically an unsolved issue. In this paper, we aim to develop better volatility prediction models using the state-of-art artificial intelligence (AI) techniques, which may shed some light on this issue.

**Fig 1:**
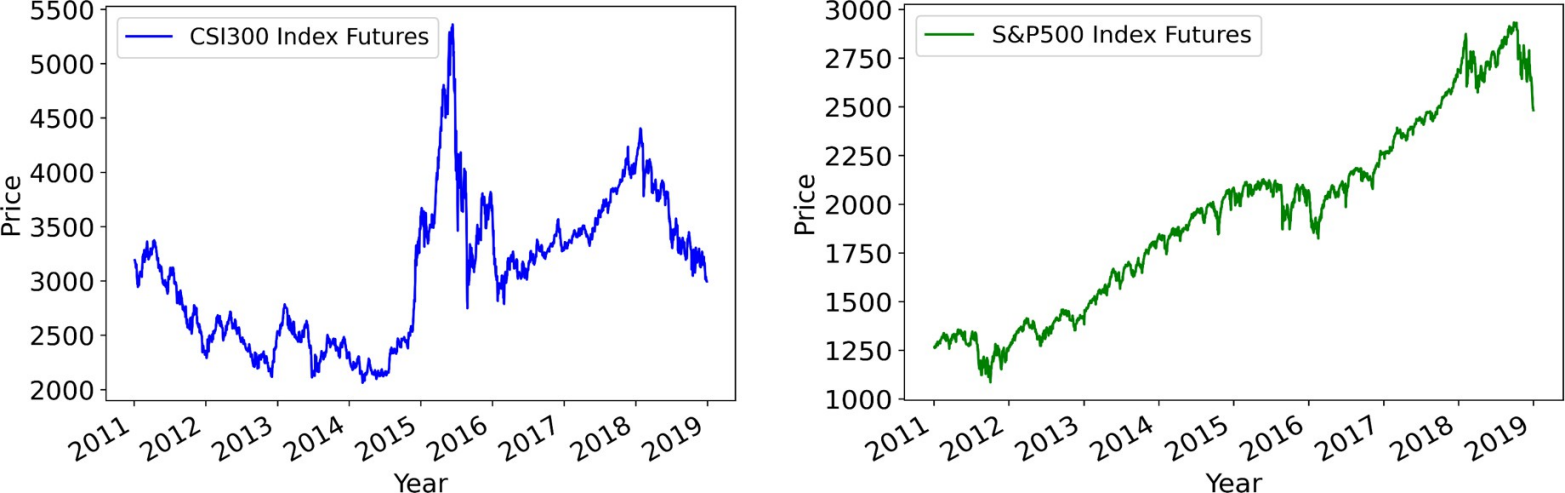
The price dynamics of CSI300 and S&P500 index futures.

To this end, we develop and compare several models for stock index volatility prediction based on the synergic use of AI techniques, such as artificial neural network (ANN), long short-term memory (LSTM) network, and auto-encoder (AE). In financial industry, two well-known conventional approaches to predict the volatility are the autoregressive (AR) model and the generalized autoregressive conditional heteroskedasticity (GARCH) model [1]. However, the applicability of the AR and GARCH models relies critically on the quality of data. If the price movements are quite irregular or erratic–for instance, the dynamics of the CSI300 index futures in China (a typical premature financial market), see Fig 1 –the prediction results of the AR and GARCH models cannot be very satisfactory. Fortunately, the recent advancement of the AI techniques, characterized by the so-called deep learning methodology, opens up new possibilities for us to better forecast volatility. We will explore these possibilities by developing a variety of hybrid AI models and assessing their prediction performances.

To assess the effectiveness of the proposed AI models, AR and GARCH models are utilized as benchmarks. In fact, the GARCH model is quite popular among industrial practitioners ever since Bollerslev [2], which generalized the autoregressive conditional heteroscedasticity (ARCH) model firstly introduced by Engle [3]. One merit of the GARCH model is that they can capture volatility clustering and leptokurtosis in financial time series [4]. Now, a variety of GARCH-type models have derived from the standard GARCH model, such as TARCH (threshold ARCH), EGARCH (exponential GARCH), FGARCH (fractional GARCH), and so on. Among them, EGARCH is quite popular. It is proposed by Nelson [5]. As a classic econometric model, EGARCH is widely utilized and demonstrates great fitting and predicting performance in finance [6–10]. The reason may be that it allows negative shocks to have a distinct impact upon volatility than positive impacts, consequently it can capture the commonly observed asymmetries of volatility in finance. Our regression results support the findings of Nelson [5]. Thus, the EGARCH model is applied as a benchmark for volatility forecast.

ANN model is designed to mimic the knowledge-acquisition and organizational skills of human brain [11, 12]. In the field of financial time series, Ormoneit and Neuneier [13] used a multilayer perceptron and density-estimating neural network to predict the volatility of the DAX German index. Later, Hamid and Iqbal [14] and McAleer and Medeiros [15] also used an ANN model to predict the volatility of S&P500 index futures. There are some other recent studies on financial market volatility using ANN models [16–19]. Other applications of ANN and some comparisons between ANN and conventional statistical models can be found in Özkan [20], Claveria and Torra [21], Mostafa and El-Masry [22], Qiu and Song [23], Pyo et al. [24], and references therein.

Deep learning, which has its root in ANN, is a state-of-art AI technique that enables computers to learn from data via repeated training. Typical examples applying deep learning technique include image recognition [25], language translation [26, 27], and the AlphaGo [28]. Among them, LSTM is widely used in sequential data prediction. It was firstly proposed by Hochreiter and Schmidhuber [29], and has feedback connections inside the neural network to learn temporal patterns.

LSTM is widely applied in predicting financial time series, and scholars still aim to construct new hybrid LSTM models nowadays in order to extract more useful information from real data, so as to improve the prediction performance. Maknickienė and Maknickas [30] applied a LSTM model to predict exchange rates and foreign exchange trading. Later, Bao et al. [31], Nelson et al. [32], Kim and Kim [33] also used the LSTM model to predict future trends of stock prices. Recently, some studies have combined the LSTM model with other models to make predictions. For example, Kim and Won [34] combined the LSTM model with various GARCH-type models to forecast the volatility of KOSPI200 stock index. Hu et al. [35] integrated LSTM and ANN networks with GARCH model to predict copper volatility. Vidal and Kristjanpoller [36] proposed a hybrid CNN-LSTM model to forecast gold volatility. Ji et al. [37] used a novel improved particle swarm optimization (IPSO) method with LSTM to predict stock price, of which the parameters are optimized by improved optimization algorithms [38]. This paper further extends this field. In order to improve the quality of the data that is put into the neural network, both AE and PCA are utilized as data preprocessing methods. In doing so, the proposed hybrid LSTM(AE) and LSTM(PCA) methods can be better trained and then obtain better prediction power.

AE is an unsupervised deep learning method to extract information from raw data [39, 40]. From a different angle, AE can also be perceived as data processing method for the purpose of dimensionality reduction. In this sense, one may draw some parallels between AE and principal component analysis (PCA), which is another popular dimensionality reduction method [41, 42]. However, the difference is that PCA is a linear method while AE is usually nonlinear [43]. Still, some researchers suggest that it is possible to stack the AE layer by layer to extract more abstract information from the data [44]. In this study, we integrate ANN and LSTM with both PCA and AE, which are both important methods to extract useful information.

The main contributions of this study are as follows. First, we propose a novel method to apply PCA to extract useful information from stock index and futures trading data in order to better fit the LSTM network to predict volatilities of stock index futures in China and the US. Second, six AI models and two statistical models are carefully investigated and compared. The empirical results find that the proposed LSTM(PCA) model shows better volatility prediction performance than the other models. Third, we further provide a discussion on the model choice between ANN(PCA) and LSTM(PCA), both of which are found to outperform other models in certain cases. It seems that LSTM can capture more useful information from more complex financial time series data, thus it can obtain better volatility prediction results than the normal ANN model in the Chinese and US stock markets.

The remainder of this paper is structured as follows. In section 2, we introduce the eight prediction models. In section 3, we conduct the empirical analyses to assess the prediction performances of various models. Section 4 summarizes the main results and concludes this study.

## 2. Methodology

### 2.1 Prediction models

#### AR model

AR model is a simple but classical linear predictive modeling technique, which uses the historical data to make predictions. When only one lagged value *x*_*t*−1_ is useful in predicting *x*_*t*_, we get the AR(1) model. It is defined as:

$$x_{t}=\beta_{0}+\beta_{1}x_{t-1}+\varepsilon_{t}$$ (1)

#### EGARCH model

GARCH model, proposed by Bollerslev [45], is a classic type of statistical models for time series data, which describes the variance of the current error term as a function of the error terms in previous period. In finance, the EGARCH model (i.e. exponential GARCH model) is widely used to describe the dynamic behavior of the volatility of financial time series, which are usually subject to asymmetric impacts of negative and positive shocks [5]. The specification for the EGARCH (1,1) model is defined as:

$$R_{t}=\theta^{'}X+\varepsilon_{t}$$ (2)

$$\ln\sigma_{t}{}^{2}=\alpha +\beta \ln\sigma_{t-1}^{2}+\gamma \frac{\varepsilon_{t-1}}{\sigma_{t-1}}+\phi |\frac{\varepsilon_{t-1}}{\sigma_{t-1}}|$$ (3)

Eq (3) guarantees that the conditional variance is positive and there is no need to restrict the parameters to be nonnegative. The impact is asymmetric if *ϕ*≠0.

#### ANN model

**Fig 2:**
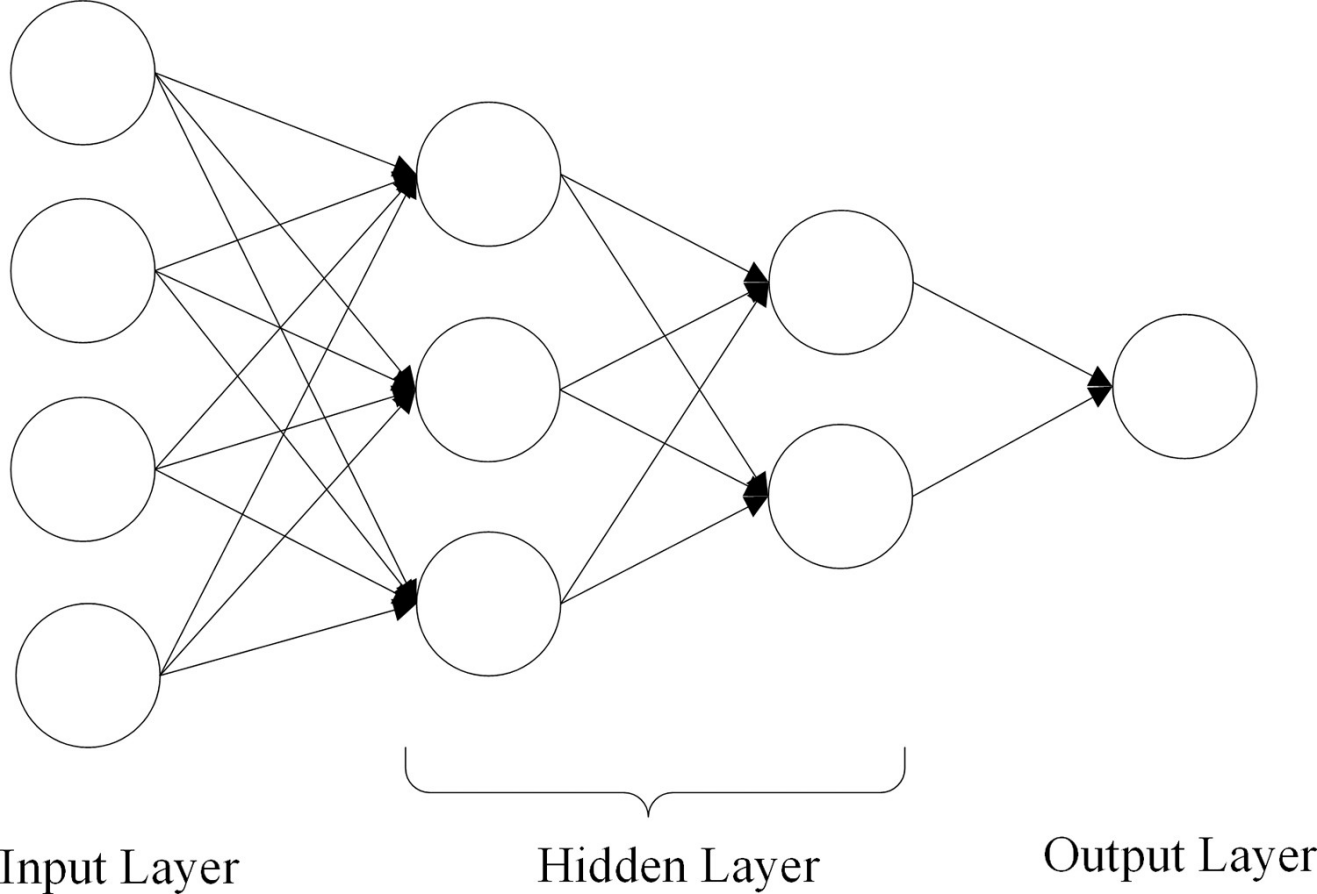
An illustrative structure of ANN.

ANN is a kind of neural network model inspired by the biological neural networks of human brains. It receives inputs, changes their internal states based on the inputs, and finally calculates the outputs. The weights of these artificial neurons can be updated through a process called learning. One simple kind of ANN architecture is demonstrated in Fig 2. It uses the output of the previous layer as the input of the current layer, and sequentially computes the values of each layer, and then gets the final value from the output layer.

The formulation about the ANN model showed in Fig 2 is as follows:

$$y=f(f(f(xw_{xj}+b_{j})w_{jk}+b_{k})w_{kl}+b_{l})$$ (4)

In Eq (4), *f*(.) is the activation function. The input variables *x* is multiplied by weight *w*_*xj*_ and summed with bias *b*_*j*_. The inputs of one layer is based on the results of the previous layer, the final output *y* is the predicted value.

#### LSTM model

**Fig 3:**
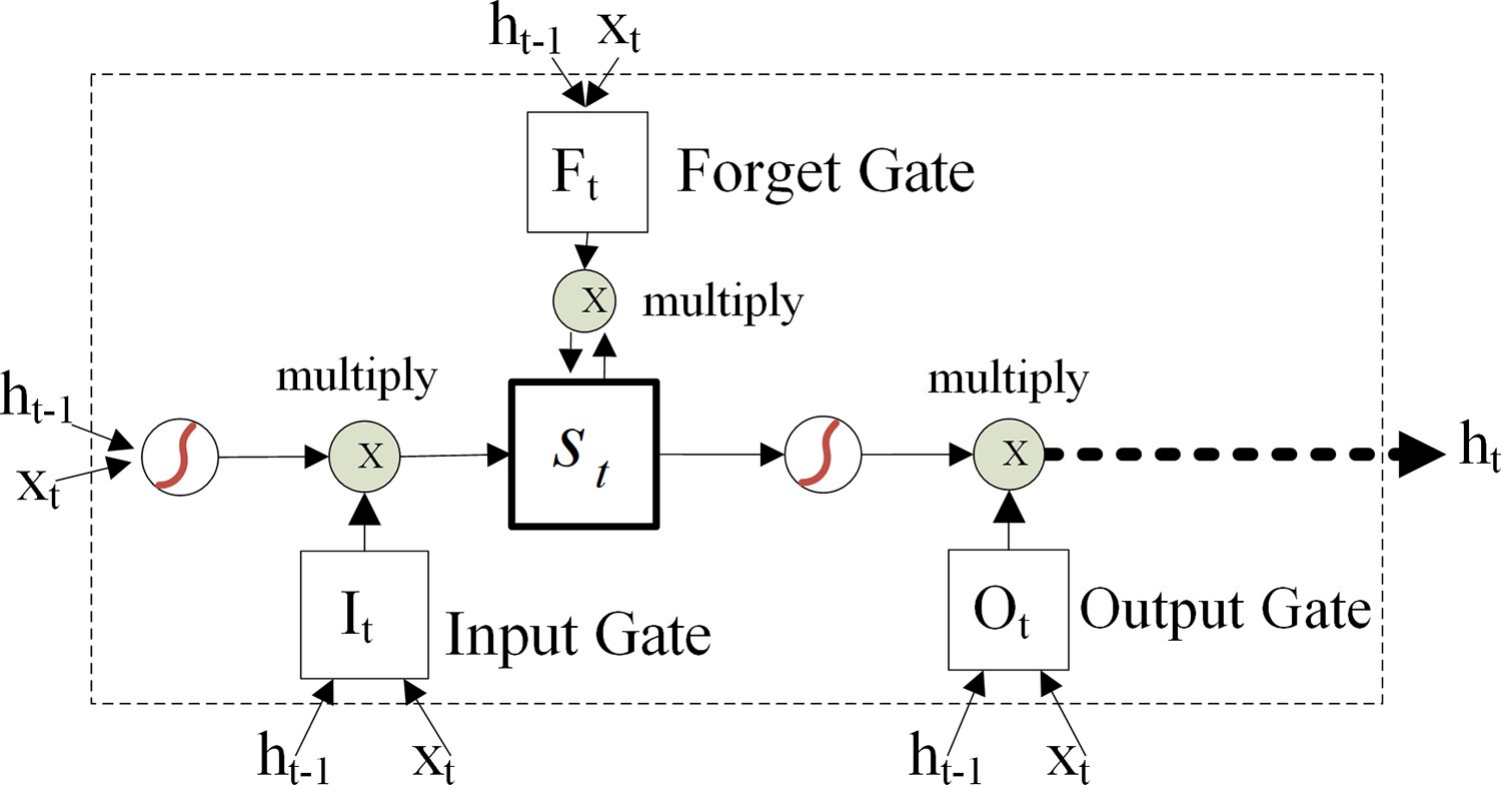
An illustrative structure of a LSTM unit.

LSTM is a classic type of recurrent neural networks (RNNs). Unlike the previous memory-free ANN model, LSTM can learn tasks that require a long-term memory for events. LSTM can handle with the problem of explosion and disappearing gradient which may be encountered when training traditional RNNs. Fig 3 shows the structure of a LSTM unit, which illustrates that the LSTM contains a memory cell (*S*_*t*_), and three gates: an input gate (*I*_*t*_), a forget gate (*F*_*t*_), and an output gate (*O*_*t*_). For more information about LSTM, please see Hochreiter and Schmidhuber [29] and Gers et al. [46].

The equations for a LSTM unit are given in (5)—(9):

$$i_{t}=\sigma (w_{ix}x_{t}+w_{ih}h_{t-1}+b_{i})$$ (5)

$$f_{t}=\sigma (w_{fx}x_{t}+w_{fh}h_{t-1}+b_{f})$$ (6)

$$o_{t}=\sigma (w_{ox}x_{t}+w_{oh}h_{t-1}+b_{o})$$ (7)

$$s_{t}=f_{t}⊗s_{t-1}+i_{t}⊗\tanh(w_{sx}x_{t}+w_{sh}h_{t-1}+b_{s})$$ (8)

$$h_{t}=o_{t}⊗\tanh(s_{t})$$ (9)

Eqs (5)–(8) shows the formulation for input gate (*i*_*t*_), forget gate (*f*_*t*_), output gate (*o*_*t*_), and memory cell (*s*_*t*_). *σ*(.) is the sigmoid function and tanh(.) is the hyperbolic tangent function. In these equations, *x*_*t*_ is the input vector at time *t*, and *h*_*t*_ denotes the hidden state vector which is also known as the output vector, the operator ⊗ represents the element-wise product (i.e. Hadamard product). The initial values are *s*_0_ = 0 and *h*_0_ = 0. Besides, *w* and *b* are weight matrix and bias vector respectively, which need to be learned during training.

#### Features extraction methods

In this study, both PCA and AE are used to extract useful information. PCA is one of the most popular unsupervised linear techniques to transform high-dimensional data into a lower dimensional one [47]. The purpose of PCA is to construct a low-dimensional representation of the raw data while keeping the maximal variance and covariance structure of the data. AE is an artificial neural network which also abstracts representations of the data in an unsupervised manner. Such representations in AE can be very informative. Since AE can compress data significantly while retaining useful information, it is also used for the purpose of dimensionality reduction. Please see Liou et al. [39, 40] and Vincent et al. [44] for more technical details of AE. The difference between AE and PCA is that PCA is a linear method while AE is usually nonlinear.

Based on the aforementioned statistical and AI models, we apply eight different prediction models to forecast the price volatilities of the stock index futures in China and the US. These models are as follows. 1) AR: a classic statistical model for financial time series. It attempts to predict future values based on previous observations. It serves as a benchmark to assess the effectiveness of the proposed prediction models. 2) EGARCH: another classic statistical model for financial time series. It incorporates the leverage effect to reflect the asymmetric impacts of negative and positive shocks, and also serves as a benchmark. 3) ANN: a conventional neural network model designed to mimic the knowledge-acquisition and learning process of human brain. It is commonly used in literature to forecast volatility. 4) ANN(PCA): an extension of the ANN model, it uses the PCA method for data preprocessing before the supervised training of the ANN network. It can reduce the dimensionality of the inputs to the ANN network and thus increase the efficiency of model training. 5) ANN(AE): another extension of the ANN model, it uses an AE network which is firstly trained in an unsupervised manner for data preprocessing before the supervised training of the ANN network. The use of the AE method is to obtain compressed and abstract representations of the data that contains key information from the data. 6) LSTM: a typical type of the RNNs, it has feedback connections inside the neural network to store information for long periods of time. 7) LSTM(PCA): an extension of the standard LSTM model, it uses the PCA method for data preprocessing before the supervised training of the LSTM network. It can reduce the dimensionality of the inputs to the LSTM network and thus increase the efficiency of the model training. 8) LSTM(AE): another extension of the LSTM model, it uses an AE network for data preprocessing before the supervised training of the LSTM network. The use of the AE method is to obtain compressed and abstract representations of the data that contains key information from the data.

### 2.2 Data preprocessing

#### 2.2.1 Data standardization

Before the training of neural networks, it is very important to standardize the input values. The standardization can enhance the training efficiency of the neural networks. The widely used MinâMax method is applied in this study.

$${\hat{x}}_{t}=\frac{x_{t}-x_{\min}}{x_{\max}-x_{\min}}$$ (10)

where *x*_max_ and *x*_min_ are the maximum and minimum value of variable *x*, respectively. *x*_*t*_ is the actual value and ${\hat{x}}_{t}\in [0,1]$ is the standardized value on day *t*.

#### 2.2.2 Return calculation

The returns of stock index futures on day *t* are computed as follows:

$$R_{t}=(\frac{P_{t}}{P_{t-1}}-1)*100$$ (11)

where *P*_*t*_ and *P*_*t*−1_ are the closing prices of the stock index futures on day *t* and *t*−1, respectively.

#### 2.2.3 Volatility calculation

Volatility plays a very important role in the financial market and it often refers to the level of uncertainty or risk related to the asset [48]. In this paper, we try to predict the volatility of stock index futures through several neural network models. The realized volatility (*RV*_t_) on day t during *T* days is calculated as follows:

$$RV_{t}=\frac{1}{T}\sum_{i=t+1}^{i=t+T}{\left(R_{i}-\bar{R}\right)}^{2}$$ (12)

where *R*_*i*_ is return of the stock index futures on day *i*. And *T* is the number of days after day *t*. $\bar{R}$ and *RV*_t_ are the average return and realized volatility of the stock index futures during *T* days, respectively.

#### 2.2.4 Loss function

To evaluate and compare the performances of various prediction models, three of the most common metrics, i.e., the mean squared error (MSE), the mean absolute error (MAE), and the normalized mean squared error (NMSE), are used. MSE is the mean of the square of the difference between the actual and predicted values. MAE denotes the average of absolute deviation of the predicted values from the actual ones. NMSE, which is a normalization of the MSE, is another estimate of total deviation between predicted and actual values. They are defined as follows:

$$MSE=\frac{1}{N}\sum_{t=1}^{N}{\left(RV_{t}-FV_{t}\right)}^{2}$$ (13)

$$MAE=\frac{1}{N}\sum_{t=1}^{N}\left|FV_{t}-RV_{t}\right|$$ (14)

$$NMSE=\frac{\sum_{t=1}^{N}{\left(RV_{t}-FV_{t}\right)}^{2}}{\sum_{t=1}^{N}{\left(RV_{t}-\bar{V}\right)}^{2}}$$ (15)

where *FV*_*t*_ and *RV*_t_ are the forecasted and realized volatilities of the stock index futures, respectively, *N* is the number of predictions. $\bar{V}$ is the historical mean value of the realized volatilities. For MSE, MAE, and MAPE, lower values indicate better predictions.

### 2.3 The procedure of volatility prediction

**Fig 4:**
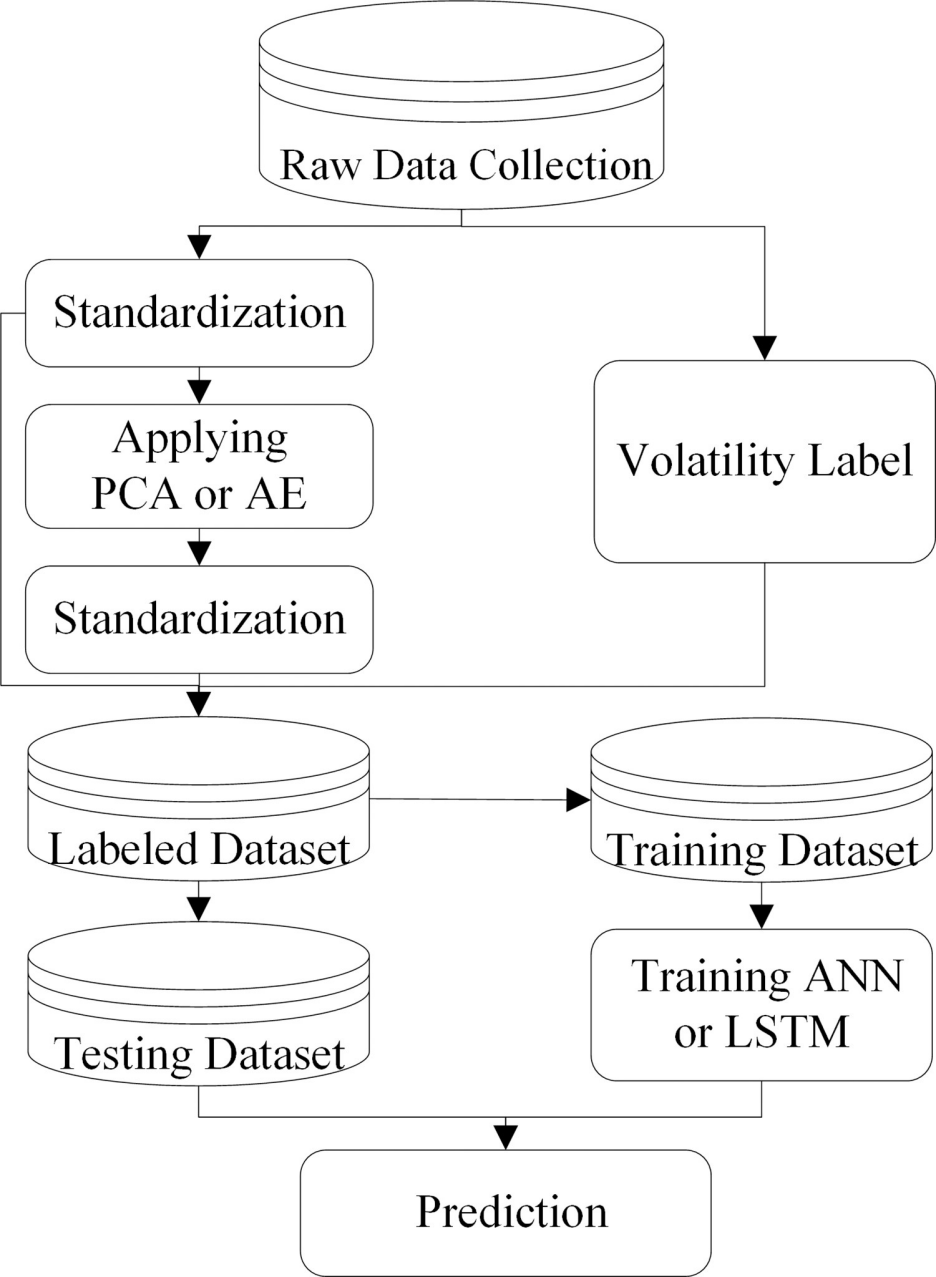
The procedure of the volatility prediction scheme using ANN or LSTM.

As illustrated in Fig 4, The procedure of volatility prediction in this study consists of three basic phases: (1) inputting data selection, raw data collection, missing values processing. (2) standardization, applying PCA and AE to extract influential features, adding labels. (3) partitioning labeled dataset into training and testing datasets, training the ANN and LSTM models, predicting the volatility of stock index futures.

## 3. Empirical analysis

### 3.1 Data description

In this section, the efficacies of the proposed prediction models in forecasting the return volatility of stock index futures in China and the US are investigated. We focus on the nearby futures contracts which are highly liquid in the stock index futures market. Eight different prediction models, namely, AR, EGARCH, ANN, ANN(PCA), ANN(AE), LSTM, LSTM(PCA), and LSTM(AE), are compared.

The dataset in China that we use includes the daily closing prices of CSI300 index futures, as well as seven major stock indices in China, namely, CSI300 index, CSI500 index, SSE composite index, SZSE component index, SZSE composite index, Chinext price index, and Chinext composite index. The latter seven stock indices can be regarded as relevant features that can improve the accuracy of the prediction models for CSI300 index futures. In addition, two commodity futures, crude oil and gold futures, are also included in the dataset. The data are collected from January 1, 2011 to December 31, 2018. The data source is the Wind database. The US dataset utilized in this study includes the daily closing prices of S&P500 index futures, as well as six major stock indices in the US, namely, S&P500 index, NASDAQ index, NASDAQ-100 index, Dow Jones Global index, Dow Jones Americas index, and S&P500 VIX, which can be regarded as relevant features that can improve the accuracy of the prediction models for S&P500 index futures. In addition, crude oil and gold futures are also included in the dataset. The data are also collected from January 1, 2011 to December 31, 2018. The detailed description of the datasets is given in Table 1.

**Table 1:** Summary of the datasets in China and the US.

Group	Name	Code
**China**	CSI300 index futures	IF00.CFE
	CSI300 index	399300.SZ
	CSI500 index	000905.SH
	SSE composite index	000008.SH
	SZSE component index	399001.SZ
	SZSE composite index	399106.SZ
	Chinext price index	399006.SZ
	Chinext composite index	399102.SZ
	crude oil futures	T00.IPE
	gold futures	GC00.CMX
**US**	S&P500 index futures	SP00.CME
	S&P500 index	SPX.GI
	NASDAQ index	IXIC.GI
	NASDAQ-100 index	NDX.GI
	Dow Jones Global index	W1DOW.GI
	Dow Jones Americas index	DJUS.GI
	S&P500 VIX	VIX.GI
	crude oil futures	T00.IPE
	gold futures	GC00.CMX

For both the Chinese and US datasets, missing values are filled with the last valid values of previous trading days. Then, each dataset is sequentially divided into two sets, i.e., the training set which contains 70% of the data, and the testing set which contains the remaining 30% data. After some initial tests, a 20 trading days window (approximately four weeks) is used to forecast both the 5 days ahead and 10 days ahead volatilities. To facilitate the empirical analysis, in what follows, all returns are scaled up by a factor of 100, and so all variances are scaled up by a factor of 10000.

Table 2 summarizes the common descriptive statistics. It is shown that the distributions of CSI300 and S&P500 index futures returns reveal negative skewness and high kurtosis, and the Jarque-Bera tests reject the normal distribution hypothesis. Besides, as to the volatility, the statistical values (i.e., mean, standard deviation, skewness, and kurtosis) of CSI300 index futures are much larger than those of S&P500 index futures.

**Table 2:** Summary statistics of CSI300 and S&P500 index futures.

	Mean	Std. Dev.	Skewness	Kurtosis	Jarque-Bera	P-value
**CSI300 Return**	0.0103	1.6451	-0.33	12.25	1740.04	0.000
**S&P500 Return**	0.0382	0.9010	-0.34	7.50	87.25	0.000
**Var5 of CSI300**	2.2154	5.1823	7.57	80.08	493864.70	0.000
**Var10 of CSI300**	2.4590	4.8006	6.00	46.94	165625.20	0.000
**Var5 of S&P500**	0.6623	1.0964	4.41	29.13	63151.69	0.000
**Var10 of S&P500**	0.7394	0.9751	2.91	12.86	10865.99	0.000

Note: Var5 represents 5-day volatility while Var10 indicates 10-day volatility.

### 3.2 Empirical performances of different prediction models

We now examine the performances of the eight prediction models, which include two traditional statistical model (AR and EGARCH), three forward artificial neural network models (ANN, ANN(PCA), and ANN(AE)), and three recurrent network models (LSTM, LSTM(PCA), and LSTM(AE)). Each of these network models is trained using the training set via the stochastic gradient descent (SGD) algorithm with 50 training epochs. The prediction performances of the trained models are assessed using the corresponding testing sets. For the 5 days (or 10 days) ahead volatility prediction tasks, the results are labeled as 5 days (or 10 days) ahead volatilities of the CSI300 and S&P500 index futures. Table 3 summarizes the prediction performances in terms of MSE for predicting the 5 days ahead and 10 days ahead volatilities for the CSI300 and S&P500 index futures.

**Table 3:** Prediction performances in terms of MSE on testing sets.

	CSI300 index futures		S&P500 index futures	
	5 days ahead	10 days ahead	5 days ahead	10 days ahead
**AR**	2.6111	2.2550	1.4002	0.8453
**EGARCH**	14.1929	11.0698	1.4130	0.9461
**ANN**	7.9999	8.1771	1.1763	0.7378
**ANN(PCA)**	14.7999	7.7900	1.1884	**0.7355**
**ANN(AE)**	4.4246	5.9874	1.3116	0.8548
**LSTM**	2.2472	2.1899	1.2254	0.8512
**LSTM(PCA)**	**1.9759**	**1.8998**	**1.1280**	0.8239
**LSTM(AE)**	2.2507	2.2992	1.2354	0.8285

First, we analyze the performances of different prediction models for the 5 days ahead volatility of CSI300 index futures. Table 3 shows that the MSE of LSTM(PCA) is 1.9759, while the lowest MSE that the other two LSTM-type models can achieve is 2.2472. Thus, LSTM(PCA) is the best LSTM-type model in this case. In addition, the best ANN-type model is ANN(AE), the MSE of which is still 4.4246, meaning that the ANN-type model is weaker than the LSTM-type models in predicting the 5 days ahead volatility for the CSI300 index futures. Moreover, AR shows a MSE of 2.6111, which is larger than MSE values of the LSTM-type models. Further, the MSE of EGARCH is 14.1929, which is significantly larger than both the ANN-type and LSTM-type models. We then analyze the results of the 10 days ahead volatility prediction for CSI300 index futures. From Table 3, we observe that LSTM(PCA) still has a lowest MSE of 1.8998, which is significantly smaller than the MSE (2.1899) of LSTM and the MSE (2.2992) of LSTM(AE), and is also substantially lower than the MSE values of the three ANN-type models, and the MSE values of AR and EGARCH models. From the first two columns of Table 3, we can conclude that the LSTM(PCA) consistently and significantly outperforms all the other seven prediction models in predicting the volatility of CSI300 index futures in China.

We now turn to investigate the performances of these volatility prediction models on S&P500 index futures. We examine the 5 days ahead prediction results firstly. From Table 3, we can see that the MSE of LSTM(PCA) is 1.1280 while the MSE values of the other two LSTM-type models are no smaller than 1.2254 and the MSE values of the ANN-type models are no smaller than 1.1763. Although both the LSTM-type and ANN-type models can achieve lower MSEs than the EGARCH model (with a MSE of 1.4130) and the AR model (with a MSE of 1.4002), LSTM(PCA) still demonstrates the best prediction performance, which is in line with the results of the CSI300 case. However, it’s found that some LSTM-type models cannot outperform the ANN-type models, e.g., the LSTM has a MSE of 1.2254, which is slightly higher than the MSE (1.1763) of ANN. This result is in contrast with the CSI300 case, where all the LSTM-type models are significantly better than the ANN-type models.

We finally examine the model performances in predicting the 10 days ahead volatility of the S&P500 index futures, where a new phenomenon is observed. From the last column of Table 3, it can be found that ANN(PCA) has a MSE of 0.7355, which is not only the lowest among the three ANN-type models, but also marginally better than all the three LSTM-type models (The MSE of LSTM(PCA) is 0.8239, the lowest of the three LSTM-type models), and better than AR (with a MSE of 0.8453) and EGARCH (with a MSE of 0.9461) models. Further, we notice that ANN has a MSE of 0.7378, which is significantly lower than the MSE (0.8512) of LSTM.

Besides MSE, we also compare the prediction performances of various models for predicting the volatilities of the CSI300 and S&P500 index futures in terms of MAE and NMSE, and the results are shown in Tables 4 and 5, respectively. From Tables 4 and 5 jointly, we can also find that LSTM(PCA) is the best prediction model for predicting the 5 and 10 days ahead volatilities of the CSI300 index futures, and the 5 days ahead volatility of the S&P500 index futures; ANN(PCA) also dominates the other models in predicting the 10 days ahead volatility of S&P500 index futures. This implies that the results in terms of MAE and NMSE is consistent with the results according to MSE.

**Table 4:** Prediction performances in terms of MAE on testing sets.

	CSI300 index futures		S&P500 index futures	
	5 days ahead	10 days ahead	5 days ahead	10 days ahead
**AR**	1.3659	1.3282	0.5613	0.5142
**EGARCH**	3.0496	2.6699	0.5157	0.4677
**ANN**	2.4562	2.4272	0.4716	0.4695
**ANN(PCA)**	3.9114	2.3449	0.4221	**0.4245**
**ANN(AE)**	1.2986	1.8583	0.6435	0.5171
**LSTM**	1.0503	1.1503	0.4502	0.4678
**LSTM(PCA)**	**0.9622**	**1.0014**	**0.3951**	0.4711
**LSTM(AE)**	1.1620	1.2122	0.6574	0.5695

**Table 5:** Prediction performances in terms of NMSE on testing sets.

	CSI300 index futures		S&P500 index futures	
	5 days ahead	10 days ahead	5 days ahead	10 days ahead
**AR**	0.5282	0.4370	0.9459	0.8797
**EGARCH**	2.8713	2.1454	0.9545	0.9846
**ANN**	3.7698	1.5848	0.7947	0.7678
**ANN(PCA)**	3.5082	1.5098	0.8028	**0.7655**
**ANN(AE)**	0.8951	1.1604	0.8860	0.8896
**LSTM**	0.4546	0.4244	0.8278	0.8859
**LSTM(PCA)**	**0.3997**	**0.3682**	**0.7620**	0.8574
**LSTM(AE)**	0.4553	0.4456	0.8345	0.8623

Based on the above empirical results, we would like to make some comparisons with relative studies on prediction of volatility of stock markets. First, we find that the ANN-type models generally dominate the EGARCH model. D’Ecclesia and Clementi [19], Kim and Won [34] also pointed out similar results. Second, we also find that LSTM-type models outperform the EGARCH model. It is consistent with the result of Kim and Won [34]. However, this paper further extends these studies. On the one hand, AR model shows better performance than EGARCH model for realized volatility prediction. As a result, AR model should also be used as a benchmark to assess the volatility prediction power of deep learning models. On the other hand, we find that when integrated with dimensionality reduction methods especially PCA, LSTM may not always dominate ANN, which advances the results of Kim and Won [34].

We would like to provide a discussion on the model choice between ANN(PCA) and LSTM(PCA), both of which are found to outperform other models in certain cases. Specifically, ANN(PCA) is better than LSTM(PCA) only in the case of predicting the 10 days ahead volatility of the S&P500 index futures. In all the other cases, LSTM(PCA) outperforms ANN(PCA). In fact, from Table 2, the 10-day volatility data of the S&P500 index futures has the lowest standard deviation, lowest skewness, and lowest kurtosis as compared with the others (i.e., 5-day and 10-day volatilities of the CSI300 index futures, and 5-day volatility of the S&P500 index futures). This means that the 10-day volatility data of the S&P500 index futures exhibits the lowest statistical complexity. As a result, it implies that the ANN-type models only outperforms the LSTM-type models in predicting 10-day volatility of the S&P500 index futures, which shows relatively low complexity.

However, when dealing with the other three cases that show relatively high complexity, it is better to use the more complicated LSTM-type models so as to facilitate the discovery of complex patterns embedded in the data. In all, our analysis supports the use of the LSTM(PCA) model in tackling those complex stock index futures market datasets in China (i.e., the 5-day and 10-day volatilities of the CSI300 index futures). Besides, our analysis also supports the use of LSMT(PCA) in predicting 5-day volatility of S&P500 index futures in the US, which shows relatively high complexity as compared with 10-day volatility.

As a result, it seems that LSTM can capture more useful information from more complex financial time series data to obtain better volatility prediction results than the normal ANN model in the Chinese and US stock markets. We would like to provide a discussion about the possible reason. It is commonly observed in literature that the return and volatility in financial markets often exhibit considerable long-memory phenomenon [49–53]. As LSTM network contains memory cells (shown in Fig 3) that should be promising to ‘memorize’ some long-term features of the data [52]; while ANN is just a simple memory-free network. As a result, LSTM can extract more useful signals from more complex time series data to improve volatility prediction performance.

**Fig 5:**
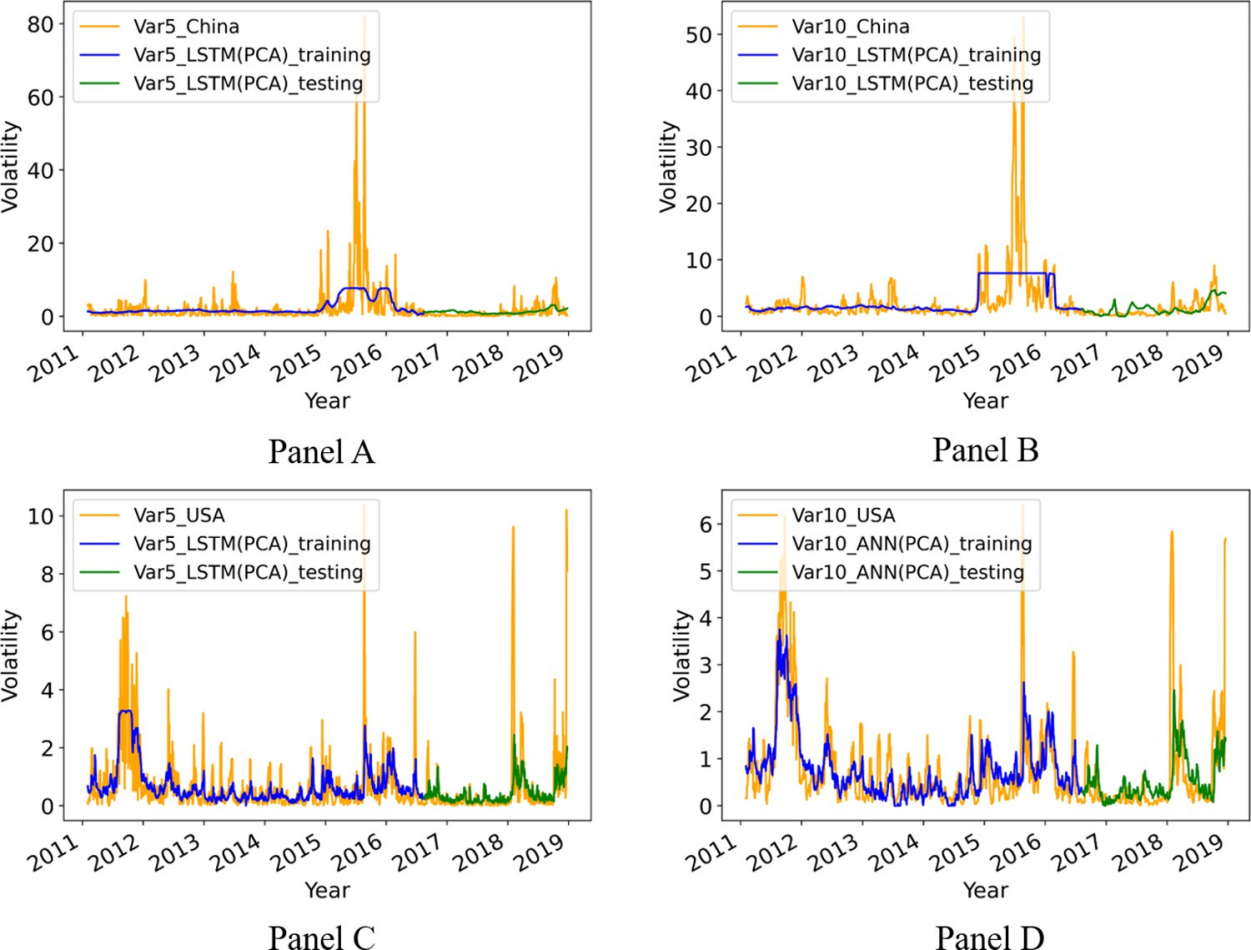
The realized and predicted volatilities of CSI300 and S&P500 index futures.

Next, we investigate the predictability of the volatilities of CSI300 and S&P500 index futures. To this end, we plot the predicted volatilities of CSI300 and S&P500 index futures against the realized ones in Fig 5. Only the best prediction models are shown. In Fig 5, Panels A, B, and C show the prediction results of LSTM(PCA) for the 5 days ahead volatility of CSI300 index futures, the 10 days ahead volatility of CSI300 index futures, and the 5 days ahead volatility of S&P500 index futures, respectively. Panel D shows the prediction result of ANN(PCA) for 10 days ahead volatility of S&P500 index futures.

From Fig 5, we notice that although the 5 days volatility of CSI300 index futures is within the range of [0, 20] in many cases, it can suddenly hike up to over 80 in some extreme cases. Similarly, the 10 days volatility of CSI300 index futures is within the range of [0, 10] in many cases, but can suddenly hike up to over 50 in some rare cases. For instance, there is a sudden stock market crash in 2015. In the first half of 2015, the stock market of China grows rapidly. Then, from June the stock market suddenly dives, as shown in Fig 1. This unexpected stock market crash greatly increased the subsequent market volatility in the second half of 2015 and the following 2016. Thus, it is understandable that it is difficult for our prediction model to catch up such a sudden hike in volatility. However, our best prediction model LSTM(PCA) can still generate a relatively high predicted volatility during 2015–2016. Although the predicted volatility is not as high as the realized volatility during that time, it can be regarded as a precautionary signal warning investors that the market volatility may hike and the risk is high. In other words, the proposed LSTM(PCA) can still be effective in producing precautionary signals that can help investors to improve risk management.

In contrast, both the 5 days and 10 days volatilities of S&P500 index futures are all within the range of [0, 10], with little extreme values, compared to that of CSI300 index futures. Thus, in a sense, the volatilities of S&P500 index futures are much more stable than that of CSI300, and so the prediction models can better predict the volatilities of S&P500 index futures.

## 4. Concluding remarks

How to develop a better volatility prediction model in finance is an important problem of both practical and theoretical interests. We have investigated the development of better prediction models on the volatility of stock index futures in two typical financial markets, including one premature market (China) and one mature market (the US). Our approach involves the synergic integration of various AI techniques (e.g., ANN, LSTM, and AE) to construct the desired effective prediction models. Specifically, we use the well-known ANN and LSTM models to account for the possible nonlinear behavior in stock market. To enhance the prediction power of the proposed models, these ANN and LSTM models are combined with two dimensionality reduction methods, i.e., PCA and AE, which can remove redundant information from the raw data. Then, using the trading data of two major index futures (i.e., CSI300 and S&P500 index futures) in China and the US, eight different prediction models including AR, EGARCH, ANN, ANN(PCA), ANN(AE), LSTM, LSTM(PCA), and LSTM(AE) are tested and compared.

We obtain the following key findings from our empirical analyses. First, all the AI models outperform EGARCH in predicting the return volatility of stock index futures. Besides, ANN(PCA) is better than LSTM(PCA) only in the case of predicting the 10 days ahead volatility of S&P500 index futures. In all the other three cases (i.e., 5 and 10 days ahead volatilities of CSI300 index futures, and 5 days ahead volatility of S&P500 index futures), LSTM(PCA) outperforms ANN(PCA). The effectiveness of PCA is generally better than AE. From the common statistics and realized volatility graphs, we find that ANN(PCA) is the best prediction model only relies on the fact that the 10 days volatility data of the S&P500 index futures exhibits the lowest statistical complexity, as compared with the other three cases. In general, our analysis supports the use of the LSTM(PCA) model in tackling those complex stock market datasets in China (e.g., the 5-day and 10-day volatilities of the CSI300 index futures), and 5-day volatility of the S&P500 index futures in the US.

From our results, one plausible guideline in developing prediction models for volatility forecasts of stock index futures in China and the US is “to use more complicated models for more complicated data”. That is, the financial market in China is premature, so the price movements are rather irregular, and so are more complicated than those in the mature market in the US. As a result, the complicate LSTM(PCA) outperformance ANN(PCA) for volatility forecasts of stock index futures in China. Moreover, in the US stock market, 5-day volatility of the S&P500 index futures shows higher level of complexity than 10-day volatility, thus the complicate LSTM(PCA) also dominates ANN(PCA). We hope that this can help shed some light on the important issue of how to accurately predict market volatility.

This paper has some limitations that can be explored in the future. For example, due to the limited resource of data, only parts of the relevant financial inputs are included in this study to increase the prediction power of the proposed model. In the future, more relevant financial and macroeconomic data may be included. Another possible extension is to examine a comprehensive comparison of various important deep learning methods for volatility forecasting, so as to further increase volatility prediction accuracy. This paper mainly focuses on constructing effective hybrid models based on the famous LSTM network. However, there exists many other important deep learning methods in financial literature, such as the customized recurrent neural network and deep reinforcement learning. This is an interesting issue that deserves research efforts in the future.

## Supporting information

S1 Dataset (RAR)
